# VivaSight™ single-lumen tube guided bronchial blocker placement for one-lung ventilation in a patient with a tracheal tumor under video-assisted transthoracic surgery: a case report

**DOI:** 10.1186/s12871-018-0677-3

**Published:** 2019-01-05

**Authors:** Jin Qiu, Miaomiao Feng, Chuanhan Zhang, Wenlong Yao

**Affiliations:** Department of Anesthesiology, Tongji Hospital, Tongji Medical College, Huazhong University of Science and Technology, Wuhan, 430030 China

**Keywords:** One-lung ventilation, Bronchial blocker, Thoracic surgery

## Abstract

**Background:**

Video-assisted transthoracic surgery (VATS) is a minimally invasive procedure that has been reported as a valid method for tracheal resection and reconstruction. However, for patients with tracheal tumors, one-lung ventilation during VATS is difficult to achieve, and utilizing a double-lumen tube is not applicable in these types of situations. When using a bronchial blocker, a fiberoptic bronchoscope is required to verify the position of bronchial blocker, though the repeated use of the fiberoptic bronchoscope increases the risk of tumor rupture and hemorrhage.

**Case presentation:**

We report a case with a middle tracheal tumor received tracheal resection and reconstruction under VATS, in which VivaSight™ single-lumen tube guided bronchial blocker placement was used for achieving one-lung ventilation. The VivaSight™ single-lumen tube can provide real-time and continuous monitoring of the position of bronchial blocker. Moreover, it does not require the aid of fiberoptic bronchoscopy.

**Conclusions:**

VivaSight™ single-lumen tube combined with a bronchial blocker is a feasible choice for one-lung ventilation in this type of surgery.

## Background

Tracheal resection and reconstruction are often used for treating malignant tracheal tumors, which are difficult procedures for both the thoracic surgeon and anesthesiologist involved. Video-assisted transthoracic surgery (VATS) is a minimally invasive procedure that has been reported as a valid method for tracheal resection and reconstruction [1, 2]. However, for patients with tracheal tumors, one-lung ventilation during VATS is difficult to achieve. A double-lumen tube (DLT) is not indicated in this situation, because it can destroy the tumor and lead to the risk of bleeding [3, 4]. When using a bronchial blocker, a fiberoptic bronchoscope is required to verify the position of bronchial blocker, though the repeated use of the fiberoptic bronchoscope increases the risk of tumor rupture and hemorrhage. VivaSight™ single-lumen tube (SLT) (ETView Ltd., Misgav, Israel) is the new generation of endotracheal tubes incorporating a high-resolution imaging camera and a light source in its tip. It can guide the placement of bronchial blocker without the aid of fiberoptic bronchoscopy. Theoretically, it makes placement of a bronchial blocker faster and provides continuous visualization. We report a case of a middle tracheal tumor in which VivaSight™ SLT guided bronchial blocker placement was used for one-lung ventilation under VATS.

## Case presentation

**Fig. 1:**
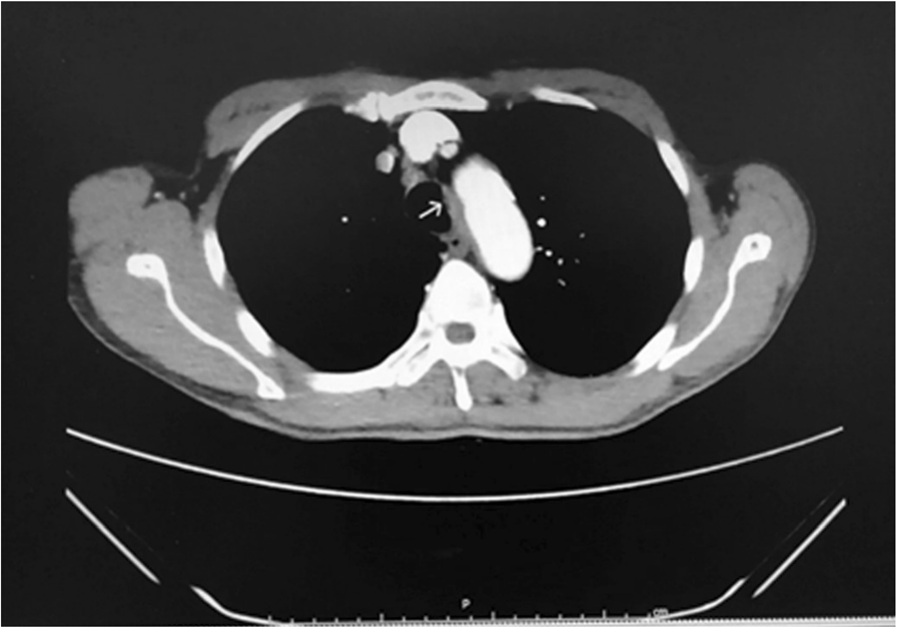
Chest computed tomography (CT) revealed a local thickening in the middle trachea

A 70-year-old, 54-kg male presented with cough and blood-stained sputum. A computed tomography (CT) scan of the chest revealed a tracheal lesion in the middle trachea (Shown in Fig. 1). Bronchoscopy showed a tumor encroaching into the left tracheal wall of the middle trachea. A biopsy was positive for an adenoid cystic carcinoma of the trachea. Routine laboratory investigations were normal. The patient’s electrocardiogram showed changes of ST-T in the anterior lateral wall and the inferior wall. Coronary CT angiography confirmed that the left anterior descending branch was mildly-to-moderately stenosed with atherosclerosis. Cardiac function was at NYHA classification level II. To avoid the stress response of open thoracotomy, the patient was scheduled for tracheal resection and reconstruction under VATS.

After arrival in the operating room, routine monitoring including electrocardiography, blood pressure, and pulse oximetry were applied. Preoperative vital signs included a blood pressure of 138/82 mmHg, heart rate of 70 beats per minute and pulse oximetry of 99%. Catheterization of the radial artery was performed under local anesthesia for continuous blood pressure monitoring as well as blood gas analysis.

**Fig. 2:**
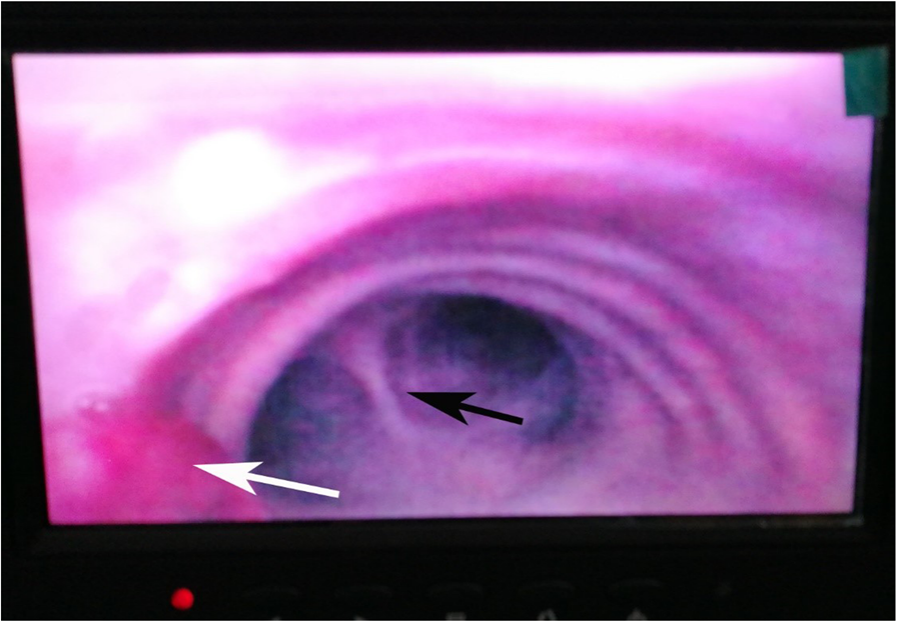
The VivaSight™ SLT was placed above the tracheal tumor under the monitor view. The white arrow refers to the tumor. The black arrow refers to carina

**Fig. 3:**
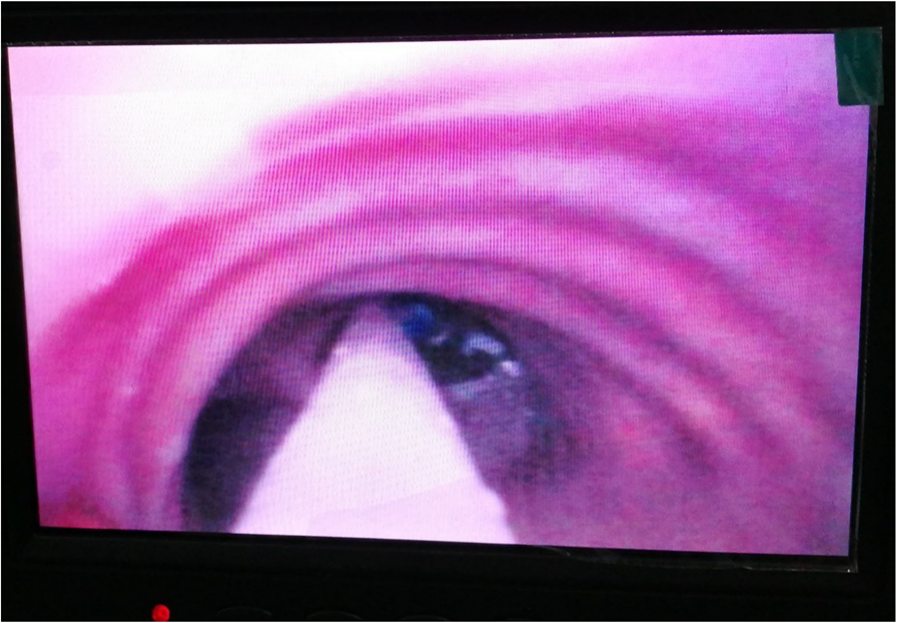
The placement of the bronchial blocker through the monitor of a VivaSight™ SLT

After preoxygenation, anesthesia was induced with intravenous sufentanil (Yichang Humanwell Pharmaceutical Company, China), propofol (AstraZeneca S.P.A., Italy), and cis-atracurium (Hengrui Medicine, Jiangsu, China). Under visual guidance of the imaging camera in the tip, an internal diameter (ID) of 7.5 mm VivaSight™ SLT was intubated through the glottis and placed above the tracheal tumor (Shown in Fig. 2). The tube was then rotated until carina and tumor were both visualized on the monitor simultaneously. A 9F bronchial blocker was placed in the right main bronchus under the guidance of VivaSight™ SLT. The cuff of the bronchial blocker was inflated with the volume necessary to seal the bronchus (8 mL air). (Shown in Fig. 3). General anesthesia was maintained with continuous intravenous infusion of remifentanil (Yichang Humanwell Pharmaceutical Company, China) 0.1–0.15 mcg•kg^− 1^•min^− 1^ and propofol (AstraZeneca S.P.A., Italy) 6–8 mg•kg^− 1^•h^− 1^. The depth of anesthesia was adjusted according to the Narcotrend index (MonitorTechnik, Bad Bramstedt, Germany). Cis-atracurium was intermittently administered to maintain muscle relaxation by the guidance of TOF-Watch (Organon, Netherlands).

The patient was placed in the left lateral decubitus and the position of the bronchial blocker was continuously monitored. Right video assisted thoracotomy was performed uneventfully to separate the trachea. Right lung collapse was achieved by deflating the cuff of the bronchial blocker and disconnecting the tube from the ventilator just before the surgeon broke the pleura. After a few seconds, the cuff of the bronchial blocker was inflated with the same volume of air and one-lung ventilation was achieved.

After the trachea was well exposed, the bronchial blocker was withdrawn, and a sterile SLT of ID 6.5 mm was placed to the left main bronchus through the incision of the trachea by the surgeon. One-lung ventilation was then performed through this tube. Following previous report [5], the lesioned tracheal was resected. During tracheal anastomosis, the patient was intermittent ventilated through the endobronchial tube, which was inserted from the incision of tracheal by the surgeon. As tracheal anastomosis was not easily performed under VATS. When the surgeon was doing the tracheal anastomosis, the SLT was removed and the ventilation was interrupted. For a while, the SLT was re-inserted and the patient was ventilated to restore oxygenation. Until the completion of tracheal anastomosis, the patient was ventilated again through the VivaSight SLT. After surgery, the patient was transferred to the thoracic ICU with a trachea tube. He was extubated at 24 h post-operatively.

## Discussion and conclusions

A different approach was selected for tracheal resection and reconstruction in this case, according to the location of the tracheal lesion. The upper part of the trachea was generally resected from the neck approach, while the procedure in the lower part of the trachea was performed from the thoracic approach [6]. With advances in minimally invasive techniques and devices, VATS resection and reconstruction of the trachea can achieve radical resection of the tumor [7]. Considering that this patient had a history of coronary atherosclerosis, VATS-guided resection and reconstruction of the trachea was chosen to reduce the stress response.

In recent years, VATS guided resection of tracheal tumors has been reported under spontaneous ventilation anesthesia [8]. The advantage of this type of procedure is that it accelerates postoperative recovery. However, the method requires that both surgeon and anesthesiologist have extensive experience with non-intubation VATS. The potential risks include hypoxemia, hypercapnia, uncontrolled coughing and soiling of the airway with blood and secretions. Therefore, we used general anesthesia with tracheal intubation in this patient.

VATS requires a clear exposure of the surgical field. DLT is routinely used to achieve one-lung ventilation, while a bronchial blocker is selected in some special situations, such as pediatric patients or difficult airways [4]. For patients with a tracheal tumor, one-lung ventilation during VATS is difficult to achieve. First, DLT is not indicated in this situation. A tumor in the trachea can hamper the placement of a DLT. Second, a bronchial blocker can be considered after an SLT is placed above the tumor. However, bronchial blockers are easy to be displaced and they require the use of the fiberoptic bronchoscope to verify its position. Furthermore, the repeated use of a fiberoptic bronchoscope can increase the risk of pulmonary infection, tumor rupture, and hemorrhage during tracheal surgery. Therefore, to minimize potential complications of fibreoptic bronchoscopy, the VivaSight SLT was used. It allows a continuous visualization of the airway and immediate correction if a displacement of the bronchial blocker occurs.

Some researchers have reported the advantages of VivaSight SLT. Karczewska et al. showed that VivaSight SLT increased the success rate of intubation at the first attempt, as compared with direct laryngoscopy [9]. Szarpak et al. claimed that VivaSight SLT may be a good first choice for tracheal intubation by a paramedic in a cervical immobilized condition [10]. Some reports showed other applications of VivaSight SLT, such as for percutaneous dilatational tracheostomy [11, 12], for novice physicians intubating without a Macintosh laryngoscope [13], for reducing the number of misplaced tracheal tubes in pre-hospital airway management [14], and for trauma patients trapped in an emergency vehicle during pre-hospital emergency care [15].

VivaSight SLT has also been reported for bronchial blocker placement without bronchoscopy. Its benefits involved a direct continuous monitoring, less time of intubation, and improved patient safety. Moritz et al. used the VivaSight SLT combined with a bronchial blocker in a case of pyothorax with bronchopleural fistula [16]. Granell et al. also demonstrated its use in an awake patient with unknown tracheal stenosis after tracheostomy in thoracic surgery [17].

In conclusion, our case demonstrates a novel approach for tracheal resection and reconstruction. VivaSight™ SLT can provide real-time and continuous monitoring of the position of a bronchial blocker and a tracheal pathological lesion. Moreover, this type of procedure did not require the aid of fiberoptic bronchoscopy, which avoided touching the tumor and interrupting ventilation. Thus, VivaSight™ SLT combined with a bronchial blocker could be a feasible choice for one-lung ventilation in this type of surgery.

## Abbreviations

CT: Computed tomography
DLT: Double-lumen tube
ID: Internal diameter
SLT: Single-lumen tube
VATS: Video-assisted transthoracic surgery
